# Supplementary figures and images for: The phenuivirus Toscana virus makes an atypical use of vacuolar acidity to enter host cells
Source: PLoS Pathog. 2023 Aug 14;19(8):e1011562. doi: 10.1371/journal.ppat.1011562 (PMC10449198; doi:10.1371/journal.ppat.1011562)

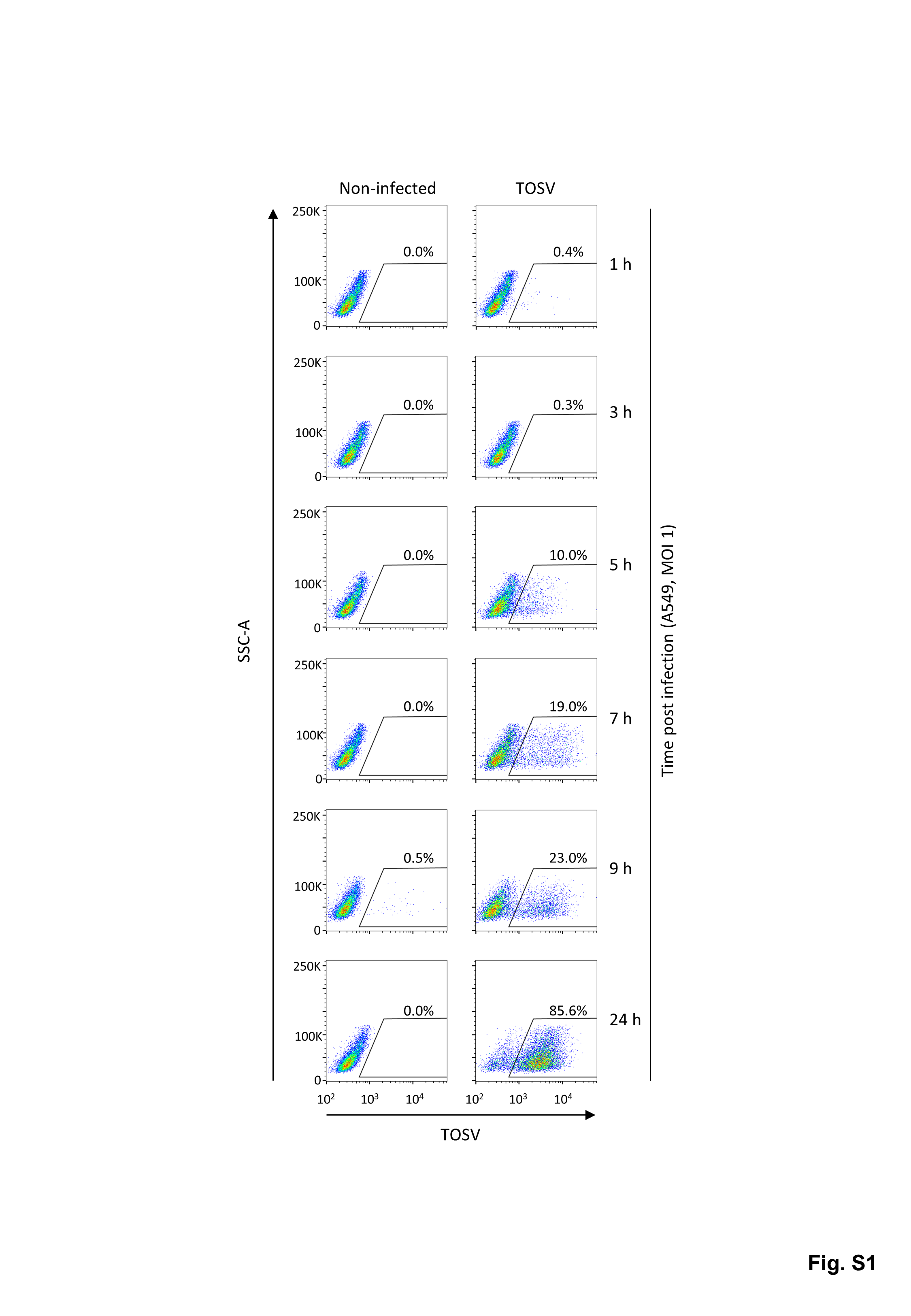

Supplement: S1 Fig — A549 cells were exposed to TOSV at MOI 1 for up to 24 h. Cells were then fixed and permeabilized, and infection was monitored by flow cytometry after immunostaining against all TOSV structural proteins, i.e., N, Gn, and Gc. (TIF) [file ppat.1011562.s001.TIF]

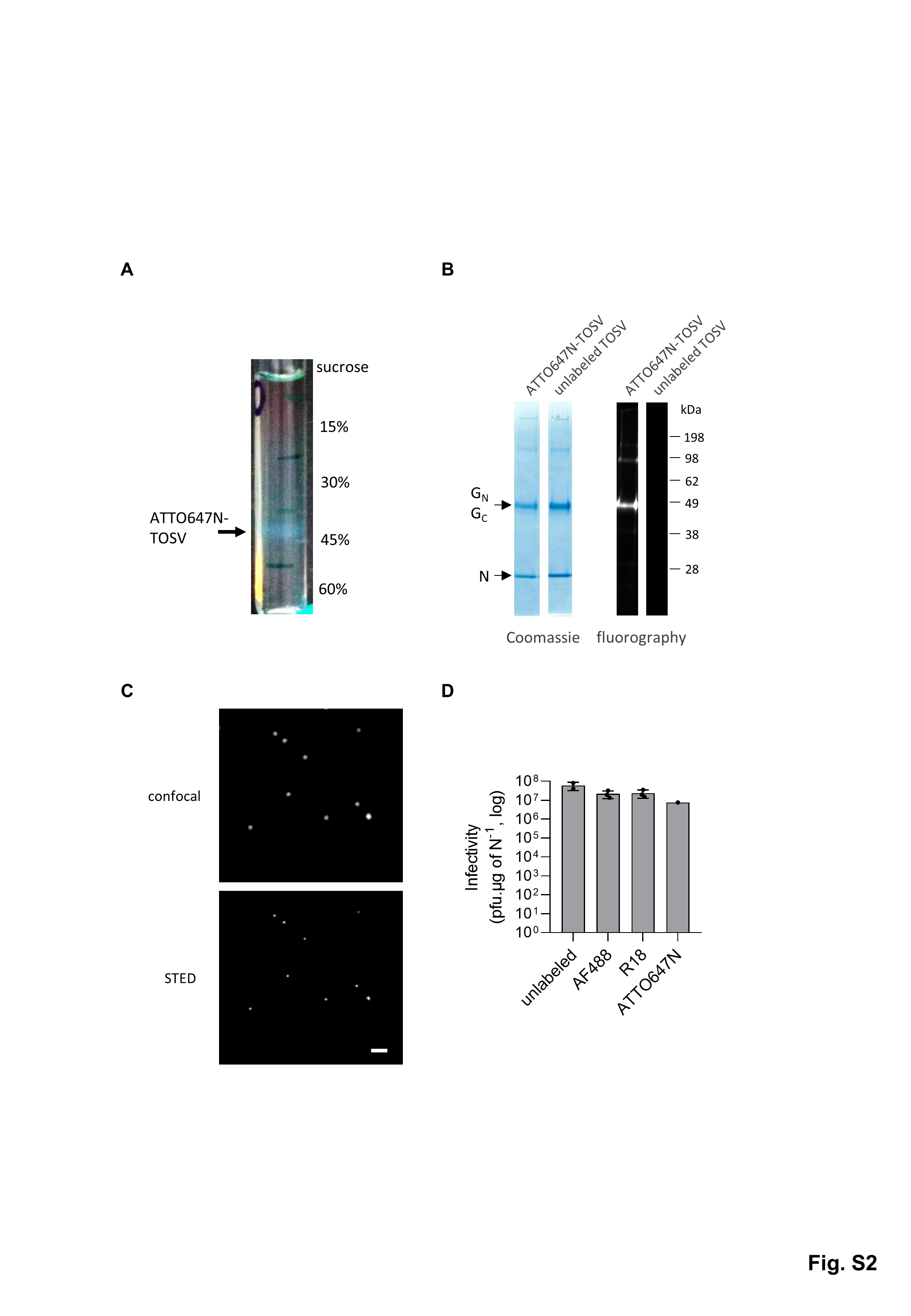

Supplement: S2 Fig — (A) The picture shows a linear sucrose gradient after ultracentrifugation with unbound ATTO647N dye on the top and a band that corresponds to ATTO647N-TOSV particles at a density between 40 and 45% sucrose. (B) Fluorescent particles (ATTO647N-TOSV) and unlabeled TOSV were analyzed by nonreducing SDS-PAGE with fluorography (right panel) and then Coomassie blue staining (left panel). (C) ATTO647N-TOSV particles were imaged by confocal microscopy (top panel) and STED microscopy (bottom panel). Scale bar, 1 μm. (D) Fluorescently labeled TOSV particles were analyzed by the pfu assay shown in Fig 1D, and the titers were normalized to the amount of the viral nucleoprotein N. (TIF) [file ppat.1011562.s002.TIF]
