# Supplementary material for: The phenuivirus Toscana virus makes an atypical use of vacuolar acidity to enter host cells
Source: PLoS Pathog. 2023 Aug 14;19(8):e1011562. doi: 10.1371/journal.ppat.1011562 (PMC10449198; doi:10.1371/journal.ppat.1011562)
Supplement: S3 Fig — The M segment of TOSV H4906 strain and RVFV 35/74 strain were aligned with EMBOSS Needle. Identical amino acids are highlighted by ‘|’, similar amino acids by ‘:’, and different amino acids ‘.’. The sequence in the M segment corresponding to NSm, Gn, and Gc were highlighted in blue, green, and yellow, respectively. (PDF) [file ppat.1011562.s003.pdf]

```
TOSV_M_H4906       1 MFIAKLLLISCLVQQAYLKYTIISEIGNGTRTSFCYSSRTSLGSLLEAWD     50

RVFV_M_35_74       1 --------------------------------------------------      0

TOSV_M_H4906      51 NVTESIGLTSGTCKVGSLENRDCRKDIMSNYVAEVHSNLHGLGAMMHNYG    100

RVFV_M_35_74       1 --------------------------------------------------      0

TOSV_M_H4906     101 DDKRVEILNDVIPDELNPGHHNCSDLDRKPFWTEFINVAKPLSEIVPMSV    150

RVFV_M_35_74       1 --------------------------------------------------      0

TOSV_M_H4906     151 IPPPVVKPQPPAEPSSKKHVPFDVLLSEAEKELNQTKIRLEDERKSNAEE    200
                                      .:|...:|::..   :.:..||:  ...|..||
RVFV_M_35_74       1 -----------------MYVLLTILITVL---VCEAVIRV--SLSSTREE     28

TOSV_M_H4906     201 TA-KERARMKEIE---DELRREWELERKRKDAMADDLAKETARRREIERK    246
                     |. .:....:.||   |.||.|         .|.::|:...:..||::..
RVFV_M_35_74      29 TCFGDYTNPEMIEGAWDSLREE---------EMPEELSCSISGIREVKTS     69

TOSV_M_H4906     247 AHELDRMLKKKERESLIAKE---------KADEDMRSHVHKP--------    279
                     :.||.|.||     ::||.:         |..||..|.|..|
RVFV_M_35_74      70 SQELYRALK-----AIIAADGLNNITCHGKDPEDKISLVKGPPHKKRVGI    114

TOSV_M_H4906     280 ----------NTTRAPAPTIITTLLMASAALA-GPLENRETNHLLNRPGN    318
                               ...|.....|..|:|.|.|..| .|:...|..||.||||.
RVFV_M_35_74     115 VRCERRRDAKQIGRETMAGIAMTVLPALAVFALAPVVFAEDPHLRNRPGK    164

TOSV_M_H4906     319 GAYTLSDFA--ESTC-TLAYGSECKSWEHLLNEELFPFFHSNLDKYSMLE    365
                     |...:....  ::|| .:.|...|.|::.||.:..||.|.|.....::||
RVFV_M_35_74     165 GHNYIDGMTQEDATCKPVTYAGACSSFDVLLEKGKFPLFQSYAHHRTLLE    214

TOSV_M_H4906     366 AATETVPILNKSSAVCTISPSTHSSNACGREASVIRKKCYSNMSAFFYIN    415
                     |..:|: |.......|.:. |.| .|.|.:|..|::..|.::..:..|:|
RVFV_M_35_74     215 AVHDTI-IAKADPPSCDLQ-SAH-GNPCMKEKLVMKTHCPNDYQSAHYLN    261

TOSV_M_H4906     416 LAGQITVVKCETNHVLSSDCGNCISKSLSG---QKVYTPVQDVFCQKGWT    462
                     ..|::..|||...:.|:.||..|  :.::|   :|...|:||:|||....
RVFV_M_35_74     262 NDGKMASVKCPPKYELTEDCNFC--RQMTGASLKKGSYPLQDLFCQSSED    309

TOSV_M_H4906     463 QSIPSTRYSKDICSIGLHTVKECKIG--TTSFERVGFIVVK-GRKMYIEQ    509
                     .........|.:|.:|:..:|:|. |  :|:.|.|.|.|.| .:|:|:::
RVFV_M_35_74     310 DGSKLKTKMKGVCEVGVQALKKCD-GQLSTAHEVVPFAVFKNSKKVYLDK    358

TOSV_M_H4906     510 MKMRSRQEYSEDQFLC------YKSESGSGSSVK-LKKFKVESCKGVTTS    552
                     :.:::.:....|.|:|      ||....||.:.: ||.|.:..|..:...
RVFV_M_35_74     359 LDLKTEENLLPDSFVCFEHKGQYKGTMDSGQTKRELKSFDISQCPKIGGH    408

TOSV_M_H4906     553 SASKCSGDEYFCSRFPCETANVEAHCILRRHSAIVEVNVNGVWVVPRCIG    602
                     .:.||:||..|||.:.|......|:|.....|.:|::.|:|||..|.|:|
RVFV_M_35_74     409 GSKKCTGDAAFCSAYECTAQYANAYCSHANGSGVVQIQVSGVWKKPLCVG    458

TOSV_M_H4906     603 YEEVLVKR-TSLKVEDTTERECDSCLWECGKNKLIVKTHGPKVVYATACS    651
                     ||.|:||| .|.|.....| .|.:|:.:|..:.|:|::.|.|:..|.||:
RVFV_M_35_74     459 YERVVVKRELSAKPIQRVE-PCTTCITKCEPHGLVVRSTGFKISSAVACA    507

TOSV_M_H4906     652 HGSCKSVMQKPATFVYLPYPGNSEIVGGDIGIHMTEESSPSNIHLVAHCP    701
                     .|.|.:..|.|:|.:.|.|||.|:..|||||:||..:....:..:|||||
RVFV_M_35_74     508 SGVCVTGSQSPSTEITLKYPGISQSSGGDIGVHMAHDDQSVSSKIVAHCP    557

TOSV_M_H4906     702 ARDSCDVSDCLFCVHGLLNYQCHTLFSALLI----STTVMSILTLLLLLV    747
                     .:|.|.|..|:.|.|||:||||||..||.::    |:..:..|.:|..::
RVFV_M_35_74     558 PQDPCLVHGCIVCAHGLINYQCHTALSAFVVVFVFSSVAIICLAILYKVL    607

TOSV_M_H4906     748 KGAKDLVKKLFHWLITPLCWLSVFCCWVVKSWKRRVGSAISRTNDTIGWR    797
                     |..|...:|    ::.||.|::||..||.|....||...|::.|..|||.
RVFV_M_35_74     608 KCLKIAPRK----VLDPLMWITVFIRWVYKKMVARVADNINQVNREIGWM    653
```

Fig. S3

```
TOSV_M_H4906      798 ENRRHAQDIERAQYTGGAPGAKYSFYGVMILGLLSSAQSCSESVIADSKI     847
                      |..:.|.. ..|.....||..:||.| :|:|.::|.|.:|||.:.|.|:|
RVFV_M_35_74      654 EGGQLALG-NPAPIPRHAPIPRYSTY-LMLLLIVSYASACSELIQASSRI     701

TOSV_M_H4906      848 MQCTTSGSSTLCKASGTVIMRLGPIGSESCLILKGLKDSEKQFISIKTIS     897
                      ..|:|.|.:|.|:.|||.::|.|.:|:|:||:|||:|:.:.:|:.|||:|
RVFV_M_35_74      702 TTCSTEGVNTKCRLSGTALIRAGSVGAEACLMLKGVKEDQTKFLKIKTVS     751

TOSV_M_H4906      898 SELTCREGESFWTTLYTPICLSSRRCHLMGECVSDRCLKWKTNQTSAEFT     947
                      |||:||||:|:||..::|.||||||||||:|||..:|||.|:.|:|||||:
RVFV_M_35_74      752 SELSCREGQSYWTGSFSPKCLSSRRCHLVGECHVNRCLSWRDNETSAEFS     801

TOSV_M_H4906      948 GKAHGDVMHENRCFEQSGGIGYGCFNVNPSCLYVHSYLKSVYRNGFKVFR     997
                      .......|.||:||||.||.|.||||||||||:||:||:||.:...:||.
RVFV_M_35_74      802 FVGESTTMRENKCFEQCGGWGCGCFNVNPSCLFVHTYLQSVRKEALRVFN     851

TOSV_M_H4906      998 CVAWNHRIRLEMTTHSRKFPMV-LMAMSTQPTDWGSIGLILDSEGITGTN    1046
                      |:.|.|::.||:|........: |.|.|::.|:|||:.|.||:|||:|:|
RVFV_M_35_74      852 CIDWVHKLTLEITDFDGSVSTIDLGASSSRFTNWGSVSLSLDAEGISGSN     901

TOSV_M_H4906     1047 SYSFMKHGSGSFAIIDDPYSPEPRKGFLGEVRCPTEETAMRASPSCKMAP    1096
                      |:||::.....:||:|:|:|..||:|||||:||.:|.:.:.|..||..||
RVFV_M_35_74      902 SFSFIESPGKGYAIVDEPFSEIPRQGFLGEIRCNSESSVLSAHESCLRAP     951

TOSV_M_H4906     1097 NLIEYQPEMDTAECTTNMIDPMAIFNRGSLPQVRDGMTFTQSIEKNTVQA    1146
                      |||.|:|.:|..|||||:|||..:|.||||||.|:..||..|.....|||
RVFV_M_35_74      952 NLISYKPMIDQLECTTNLIDPFVVFERGSLPQTRNDKTFAASKGNRGVQA    1001

TOSV_M_H4906     1147 LTTGEVKASIRLTLDDYDVVYQNSQSDCSATFKNLTGCYSCDEGSRLCYQ    1196
                      .:.|.|:|.:.|..|:::|.:..:...|.|.|.|||||||||:.|:|:|..
RVFV_M_35_74     1002 FSKGSVQADLTLMFDNFEVDFVGAAVSCDAAFLNLTGCYSCNAGARVCLS    1051

TOSV_M_H4906     1197 VKAEGETIFHFINEEESINVINKVNPGVSDYCTVLHFSRPVISIEGTYDC    1246
                      :.:.|.......|::.|::::.....|..|.|.:|||:.|.:..|..|.|
RVFV_M_35_74     1052 ITSTGTGTLSAHNKDGSLHIVLPSENGTKDQCQILHFTVPEVEEEFMYSC    1101

TOSV_M_H4906     1247 GLSKRPMVIKGTLIATAPHDDRKHEGGSSLVVNPKGGSVDFFGWLSGLTS    1296
                      ...:||:::||||||..|.|||:..||.|.||||||.||.:||.|.|||.|
RVFV_M_35_74     1102 DGDERPLLVKGTLIAIDPFDDRREAGGESTVVNPKSGSWNFFDWFSGLMS    1151

TOSV_M_H4906     1297 WLGGPLKTFLTILGFLAVGLLLVVIIIIIIRTGMQQ---ALKKKIK    1339
                      |.||||||.|.|..::|:.:.|..::|.:.|||:.:   |..||..
RVFV_M_35_74     1152 WFGGPLKTILLICLYVALSIGLFFLLIYLGRTGLSKMWLAATKKAS    1197
```

Fig. S3
