# Supplementary material for: The phenuivirus Toscana virus makes an atypical use of vacuolar acidity to enter host cells
Source: PLoS Pathog. 2023 Aug 14;19(8):e1011562. doi: 10.1371/journal.ppat.1011562 (PMC10449198; doi:10.1371/journal.ppat.1011562)
Supplement: S1 Table — aCells were infected with TOSV at MOI 1 for 18 h, fixed, permeabilized, and immunofluorescently stained against all TOSV structural proteins. Infection was quantified by flow cytometry, and the sensitivity of cells to TOSV infection (percentage of infected cells) was given as follows: +++ greater than 30%, ++ from 10% to 30%, + from 1% to 10%, ‐ less than 1%. bThe production of viral progeny was assessed by pfu titration assay and is given according to the size of plaques 72 hpi as follows: +++ greater than 1 mm, ++ from 0.5 to 1 mm, + less than 0.5 mm, ‐ no plaques. n.d., not determined. (PDF) [file ppat.1011562.s004.pdf]

Cell Line	Species	Tissue	Sensitivity to TOSV infection[a]	Production of new viral particles[b]	Original reference
A549	Human	Lung epithelial	+++	+++	(*1*)
HEK293T	Human	Embryonic kidney	+	n.d.	(*2*)
HeLa	Human	Cervix epithelial	+	+++	(*3*)
Huh-7	Human	Liver epithelial	+++	++	(*4*)
iPSC-derived neurons	Human	Neurons	++	n.d.	(*5*)
Jurkat	Human	T lymphoblast	-	n.d.	(*6*)
Raji	Human	B lymphocyte	+	n.d.	(*7*)
SUP-T1^{R5}	Human	T lymphoblast	-	n.d.	(*8, 9*)
SH-SY5Y	Human	Neuroblast	+++	n.d.	(*10, 11*)
THP-1	Human	Monocyte	-	n.d.	(*12*)
U87^{44}	Human	Glial cells	++	n.d.	(*13-15*)
BHK-21	Hamster	Kidney fibroblast	+++	+++	(*16*)
DF-1	Chicken	Embryonic fibroblast	+	++	(*17*)
L929	Mouse	Fibroblast	+	-	(*18, 19*)
MDCK	Dog	Kidney epithelial	+	++	(*20, 21*)
Vero E6	Monkey	Kidney epithelial	+++	++	(*22, 23*)
LLE/LULS40	Sand fly	Embryonic	+	n.d.	(*24*)
LLE/LULS45	Sand fly	Embryonic	+	n.d.	(*25*)
PPL/LULS49	Sand fly	Larva	++	n.d.	(*25*)

References

1. D. J. Giard *et al.*, In vitro cultivation of human tumors: establishment of cell lines derived from a series of solid tumors. *J Natl Cancer Inst* **51**, 1417-1423 (1973).
2. F. L. Graham, J. Smiley, W. C. Russell, R. Nairn, Characteristics of a human cell line transformed by DNA from human adenovirus type 5. *The Journal of general virology* **36**, 59-74 (1977).
3. W. F. Scherer, J. T. Syverton, G. O. Gey, Studies on the propagation in vitro of poliomyelitis viruses. IV. Viral multiplication in a stable strain of human malignant epithelial cells (strain HeLa) derived from an epidermoid carcinoma of the cervix. *The Journal of experimental medicine* **97**, 695-710 (1953).
4. H. Nakabayashi, K. Taketa, K. Miyano, T. Yamane, J. Sato, Growth of human hepatoma cells lines with differentiated functions in chemically defined medium. *Cancer Res* **42**, 3858-3863 (1982).
5. Y. Zhang *et al.*, Rapid single-step induction of functional neurons from human pluripotent stem cells. *Neuron* **78**, 785-798 (2013).
6. U. Schneider, H. U. Schwenk, G. Bornkamm, Characterization of EBV-genome negative "null" and "T" cell lines derived from children with acute lymphoblastic leukemia and leukemic transformed non-Hodgkin lymphoma. *Int J Cancer* **19**, 621-626 (1977).

Table S1

7. J. V. Pulvertaft, Cytology of Burkitt's Tumour (African Lymphoma). *Lancet* **1**, 238-240 (1964).
8. S. D. Smith, R. Morgan, M. P. Link, P. McFall, F. Hecht, Cytogenetic and immunophenotypic analysis of cell lines established from patients with T cell leukemia/lymphoma. *Blood* **67**, 650-656 (1986).
9. R. E. Means *et al.*, Ability of the V3 loop of simian immunodeficiency virus to serve as a target for antibody-mediated neutralization: correlation of neutralization sensitivity, growth in macrophages, and decreased dependence on CD4. *J Virol* **75**, 3903-3915 (2001).
10. J. L. Biedler, L. Helson, B. A. Spengler, Morphology and growth, tumorigenicity, and cytogenetics of human neuroblastoma cells in continuous culture. *Cancer Res* **33**, 2643-2652 (1973).
11. R. A. Ross, B. A. Spengler, J. L. Biedler, Coordinate morphological and biochemical interconversion of human neuroblastoma cells. *J Natl Cancer Inst* **71**, 741-747 (1983).
12. S. Tsuchiya *et al.*, Establishment and characterization of a human acute monocytic leukemia cell line (THP-1). *Int J Cancer* **26**, 171-176 (1980).
13. J. Ponten, E. H. Macintyre, Long term culture of normal and neoplastic human glia. *Acta Pathol Microbiol Scand* **74**, 465-486 (1968).
14. J. Fogh, W. C. Wright, J. D. Loveless, Absence of HeLa cell contamination in 169 cell lines derived from human tumors. *J Natl Cancer Inst* **58**, 209-214 (1977).
15. A. Bjorndal *et al.*, Coreceptor usage of primary human immunodeficiency virus type 1 isolates varies according to biological phenotype. *J Virol* **71**, 7478-7487 (1997).
16. I. Macpherson, M. Stoker, Polyoma transformation of hamster cell clones--an investigation of genetic factors affecting cell competence. *Virology* **16**, 147-151 (1962).
17. M. Himly, D. N. Foster, I. Bottoli, J. S. Iacovoni, P. K. Vogt, The DF-1 chicken fibroblast cell line: transformation induced by diverse oncogenes and cell death resulting from infection by avian leukosis viruses. *Virology* **248**, 295-304 (1998).
18. W. R. Earl, in *JNCI: Journal of the National Cancer Institute*. (1943).
19. K. K. Sanford, W. R. Earle, G. D. Likely, The growth in vitro of single isolated tissue cells. *J Natl Cancer Inst* **9**, 229-246 (1948).
20. I. J. Green, Serial propagation of influenza B (Lee) virus in a transmissible line of canine kidney cells. *Science* **138**, 42-43 (1962).
21. C. R. Gaush, W. L. Hard, T. F. Smith, Characterization of an established line of canine kidney cells (MDCK). *Proc Soc Exp Biol Med* **122**, 931-935 (1966).
22. E. J. Earley, K.M., The lineage of the Vero, Vero 76 and its clone C1008 in the United States. (1988).
23. P. J. Price, E. A. Gregory, Relationship between in vitro growth promotion and biophysical and biochemical properties of the serum supplement. *In Vitro* **18**, 576-584 (1982).
24. L. Bell-Sakyi, A. Darby, M. Baylis, B. L. Makepeace, The Tick Cell Biobank: A global resource for in vitro research on ticks, other arthropods and the pathogens they transmit. *Ticks Tick Borne Dis* **9**, 1364-1371 (2018).
25. L. Bell-Sakyi *et al.*, Isolation in Natural Host Cell Lines of Wolbachia Strains wPip from the Mosquito Culex pipiens and wPap from the Sand Fly Phlebotomus papatasi. *Insects* **12**, (2021).
