# Supplementary material for: The phenuivirus Toscana virus makes an atypical use of vacuolar acidity to enter host cells
Source: PLoS Pathog. 2023 Aug 14;19(8):e1011562. doi: 10.1371/journal.ppat.1011562 (PMC10449198; doi:10.1371/journal.ppat.1011562)
Supplement: Attachment — Submitted filename: Responses to Reviewers_R1_Koch.pdf [file ppat.1011562.s012.pdf]

To Peer Referee #1, #2, and #3

We would first like to thank the editor for taking the time to evaluate our work and to carefully consider the reports of the three reviewers. We are grateful for his positive feedback and for the invitation to resubmit a revised version of our study. The changes are detailed in our responses to reviewers. The figure and line numbers in our responses refer to the Figures and Word file of the revised manuscript, without any track changes. The "Materials and Methods" section and figure legends have also been updated to reflect our responses to the Reviewers. These changes are not mentioned in detail in the following responses but are visible in the version of the manuscript with the track changes option activated.

Peer Referee #1 comments

Point 1. This study examines the pathway by which Toscana virus (TOV) infects cell lines. TOV is a Bunyavirus though it has not been studied nearly as much as some other members of this virus family such as Rift Valley Fever virus. Bunyaviruses are know to be endocytosed after binding to the cell surface and are delivered to endosomes where the low pH environment induces the viral glycoproteins to under conformational changes that elicit fusion between the viral and endosomal membranes, allowing the viral genome to gain entry into the cytoplasm. These viruses have what are termed Class II fusion proteins, a number of which have been very well studied. This is a technically strong, very detailed study in which the investigators use pretty much every experimental approach that has been used to study viral cell entry pathways, from very classic approaches such as using lysosomotropic agents to elevate vacuolar pH to membrane fusion assays using fluorescent dyes and more modern assays in which mutant proteins (such as a form of Rab5) are expressed to as to interdict discrete steps in the endocytic pathway. Everything worked as expected and the results are clear and consistent: TOV like other Bunyaviruses is delivered to endosomes and undergoes low-pH dependent membrane fusion. A few comments follow.

Author response:

We first want to thank Reviewer 1 for having taken the time to evaluate our manuscript and for the positive comments.

Point 2. I don't understand the use of 'atypical' in the manuscript title. I didn't see anything that was unexpected or atypical in this series of very nice experiments. Concerned that I had missed something, I did a search for 'atypical' in the document, and other than stating that the virus makes an atypical use of vacuolar acidity, the word is not used in the results at all. The title made me think that there was something different about how this viruses enters cells, but such was not the case.

Author response:

We thank Reviewer 1 for bringing attention to this crucial matter. We agree that the reasons why the molecular and cellular mechanisms used by Toscana virus (TOSV) to enter cells are atypical were not sufficiently explained and highlighted in the original version of our manuscript. Our results indicate that TOSV Gc is a class-II fusion protein and that, consequently, TOSV belongs to the group of class-II fusion viruses. Typically, the fusion of other class-II viruses is triggered only when the surrounding pH reaches a minimum threshold value and involves irreversible mechanisms. The term "atypical" refers to the fact that TOSV differs greatly from other class II viruses in this respect. We found that TOSV fusion relies on the progressive decrease in pH along the endocytic pathway, not just on a pH threshold, and that the TOSV fusion process is highly likely reversible. To make this important point more explicit, we have reworded several sentences in the Introduction and Discussion in lines 88-92, 508-510, and 526-530, as follows:

"*We found that TOSV is a class-II fusion virus, but it differs greatly from other viruses in this class in that its fusion process is, at least in part, reversible and its activation depends on the progressive acidification within maturating endosomes, rather than a specific pH threshold. In other words, TOSV made atypical use of endosomal acidity to find its way through endosomal vesicles and enter the cytosol.*"

"*TOSV, as well as RVFV, UUKV, and GERV, evidently make atypical use of endosomal acidification in that they can remain infectious in the endocytic machinery long after acid activation.*"

"*Overall, the atypical fusion properties of TOSV and other bunyaviruses certainly confer to these viruses a remarkable adaptability to endosomal acidity and the advantage of trial and error in endocytosis pathways until they reach endosomes suitable for viral fusion, the detailed structural biology of which remains a challenge for future work.*"

Point 3. If the paper had to be trimmed, I would suggest really cutting back or even eliminating the morphological studies with labeled virions. The problem as I suspect the investigators know if the infectious particle ratio: at best it seems only 1 in 10 virions are infectious, so when looking a labeled particles by microscopy one is basically looking at non-infectious viruses that may or may not be doing the same thing as infectious viruses at early stages of infection. The experiments that immediately follow this (using inhibitors such as Rab7a) are more definitive.

Author response:

We thank Reviewer 1 for this suggestion. As we have not been asked to shorten the manuscript, we would prefer to keep the morphological study as it is important for the characterization of the fluorescently labeled viral particles. We sincerely believe that this tool can be confidently used to study TOSV entry into cells. Firstly, as Reviewer 1 points out, the results obtained with fluorescent particles are consistent with those found with the complementary approaches based on perturbants of the endocytic pathways. Most importantly, over 80% of surface-bound virus particles are internalized and competent for fusion, indicating that the vast majority of fluorescent viral particles are capable of penetrating the cytosol. Overall, these results rather point out a problem with viral replication, *i.e.*, post-entry. The explanation is certainly that most viral particles in a phenuivirus population lack one or more genomic segments, preventing them from being infectious on their own and forcing them to cooperate to achieve productive infection (Bermudez-Mendez et al., 2022, PLoS Biology). We discuss this possibility, now, in lines 424-430, as follows:

"*Confocal microscopy images showed that the number of bound virions per cell was significantly higher than the MOI. This result indicated that a low fraction of viral particles in the virus stocks were infectious, with only one virion every 10. Still, this ratio was high compared to that of other phenuiviruses, e.g., the ratio in UUKV infectivity is lower than 1:1,000 [19]. Altogether our results are consistent with a recent study showing that two distinct incomplete*

phenuivirus populations, which are unable to spread autonomously due to the lack of one or more genome segments, can cooperatively support infection and spread [36]."

Point 4. I don't think I would use the term 'rather inefficient' in describing binding to TOV to A549 cells (about 25% bound under the conditions used). I actually thought that this was rather efficient binding, but the issue is that there is no basis for comparison. Unless if the authors want to look at binding of other virions to these cells under the same conditions, I would argue that the use of subjective terms in this setting is perhaps not appropriate.

Author response:

In the Results, we reworded the title of the corresponding section and the sentence in lines 153, 157-159, and 172-173, respectively. We also changed the abstract and our discussion accordingly, in lines 22-24 and 421-422, respectively, as follows:

"*TOSV binding to cells is specific*"

"*The results showed that the fraction associated with the cells was about 25% of the virus input (Fig. 2C).*"

"*Combined, the data indicated that TOSV binding to cells is specific and likely involves one or more attachment factors or receptors.*"

"*Investigating iPSC-derived human neurons and cell lines, we found that virus binding to the cell surface was specific, and 50% of bound virions were endocytosed within 10 min.*"

"*The binding of virions to the cell surface was specific, although a minority of the total input virus was attached to cells.*"

Point 5. The acid-bypass experiments shown in Figure 5 are, I think, the correct control for the lysosomotropic experiments shown in Figure 1. At first I thought this control was missed, but it appeared later. I'ld probably re-organize things, but that is just one person's opinion and so I would just ask the authors to consider it.

Author response:

We have followed the recommendation of Reviewer 1 and reorganized Figures S1, 1, and 5 accordingly. In the new version of our manuscript, the characterization of the TOSV life cycle in neuron-like and A549 cells is now shown in Figures 1A to 1F and S1 (initially Figures 1A, 1B, and S1A to S1E). The effect of agents elevating endosomal pH on TOSV infection in neuron-like and A549 cells are now shown in a dedicated figure (Figure 5, initially Figures 1C to 1F) so that experiments involving lysosomotropic bases are followed by those showing acid-bypass results (Figure 6, initially Figure 5). The text in the Results and legends of figures has also been reorganized to reflect these changes.

Peer Referee #2 comments

Part I – Summary

Point 1. The authors report new elements concerning the mechanisms of entry and fusion of the Toscana Virus in iPSC-derived neurons and different cell lines. The manuscript is interesting and several experiments have been carried out to provide a valid hypothesis on how binding internalization and membrane fusion can occur during TOSV infection. The rationale of this study is clear; the methodology pursued in this study is accurate and well-conducted. The results are helpful to understand the mechanisms by which TOSV, as well as other members of Phleboviridae, enter and infect host cells.

Author response:

We thank Reviewer 2 for their careful reading of our manuscript and their constructive comments.

Part II – Major Issues: Key Experiments Required for Acceptance

Point 2. It is not mentioned that the virus (TOSV) was plaque-purified, and it was collected from BHK-21 infected cells. The fact that most of the infecting virus is not infectious could be due to the presence of a lot of defective particles present in the viral stock. Although it is known that virions can have even less than 3 genomic RNA segments, it would be useful to work with a rather pure viral stock. The virus was purified on sucrose gradient, but the band could be contaminated with defective particles.

Author response:

We would like to thank Reviewer 2 for suggesting plaque purification of the virus, and we will seriously consider it for our future work on TOSV infectivity and replication. Indeed, defining a defective viral particle is not an easy task. The same virus stock can exhibit a different infectious titer depending on the type of cells used for titration, meaning that viral particles that do not infect certain cells are not necessarily defective and can still infect other cells, and vice versa. However, this complex topic and fascinating debate about defective viral particles go beyond the scope of our work, and we are confident that our lab strain does not introduce any bias in the study of TOSV entry. Please refer to our response to Point 3 from Reviewer 1 for more detailed explanations.

Point 3. Discussion: the results of the experiments are sometimes reported in a not well organized way, therefore the reader may lose the thread. This is perhaps due to the amount of data the authors need to comment on. It appears that the acidic environment of the EEs is useful for the entry of TOSV into the cytosol, but LES are crucial for infection, therefore the role of both Endosomes should be better explained. I would suggest to make it clearer, trying to better schematize the path of the virus inside the cell until fusion.

Author response:

This study indeed includes a large amount of data, and most results are closely related, making it difficult to have a linear, straightforward discussion of the important findings. For the sake of clarity, the discussion has been slightly shortened and deeply reorganized to follow each step of the TOSV entry program. We now discuss successively (i) the virus attachment, (ii) the need for endocytic internalization and vacuolar acidification for virus entry, (iii) the virus intracellular trafficking, and (iv) the viral fusion and penetration into the cytosol. We also propose a graphical summary of the results in Figure 10, as requested by Reviewer 3 in their Point 2. We sincerely hope that these major changes, together with the additional explanations provided, will help readers to better follow the discussion.

Part III – Minor Issues: Editorial and Data Presentation Modifications

Point 4. - Besides 1 and 2, there are more specific references to be added describing most severe cases of neurologic infections by TOSV.

Author response:

We agree with Reviewer 2 that References 1 and 2 are not the most specific references on the clinical picture of TOSV. We have therefore replaced these two references with a more recent one in lines 47-49. This new reference describes most of the severe cases of TOSV neurological infection, *i.e.*, Ayhan and Charrel, 2020, Clin Microbiol Infect.

Point 5. -Lines 94: it would be better to specify the three sandfly cell lines used, although mentioned in Mat & Meth.

Author response:

The name of the three sand fly cell lines is now specified, in lines 110-113, as follows:

"*The three sand fly cell lines, namely LLE/LULS40, LLE/LULS45, and PPL/LULS49, allowed a complete viral cycle, but their sensitivity to virus infection was low compared to that of most of the mammalian cells.*"

Point 6. -Lines 239-240: 'Additional processing…' is a little bit confusing for the reader, since the glycoproteins have a role in the fusion.

Author response:

In some cases, acidification is not sufficient to trigger viral fusion. For instance, some enveloped viruses require their envelope glycoproteins to interact with an endosomal receptor or to undergo one or more proteolytic cleavages. This is what we mean by "Additional processing". To make it clearer, we reworded the sentences, in lines 253-255, as follows:

“*Additional processing steps were apparently not required in the endosomes to activate fusion or infection by TOSV.*”

Point 7. Line 474: please write the full form of the acronym ‘L-PVs’, as it is mentioned for the first time, and use the acronym from then on.

Author response:

The acronym “L-PVs” stands for “late-penetrating viruses” and is mentioned after its first use on line 356.

Point 8. - Is Ref. 53 missing?

Author response:

Ref. 53 (Barriga et al., 2013) was cited in the Material and Methods section, in line 592, and listed in the References section, in lines 909-912, which correspond to lines 608 and 925-928, respectively, in the new version of our manuscript.

Peer Referee #3 comments

Part I – Summary

Point 1. Koch et al. report an extensive and well-executed characterization of Toscana virus (TOSV) entry, providing alongside the data on virus morphology and predicted structural model for the fusion Gc protein. The authors developed flow cytometry, fluorescence microscopy and fluorimetry assays to characterize different stages of TOSV entry, integrating the data into a comprehensive TOSV entry model with several key points: only a small fraction of virus (~20%) efficiently binds to cells, majority of internalized virions fuse within the endosomal compartment (~80%), the fusion process is optimal at pH of 5.5 and requires passage of the virions from the early to the late endosomal (EE, LE, respectively) compartment, where the membrane fusion and release into cytosol occurs. The authors report a new and interesting observation that TOSV and two more bunyaviruses remain infectious after exposure to low pH, in contrast to most fusion proteins where the low pH triggers an irreversible conformational change of the fusion protein, rendering such virions inactivated. The novelty of this manuscript relies in the studied object, the TOSV, which is a re-emerging human pathogen in south Europe and north Africa, which has been poorly investigated and characterized. The findings presented here therefore fill an important gap in knowledge related to the virus that is responsible for a growing number of neuro-invasive infections that lead to meningitis in most severe cases. The data in the manuscript are presented in a clear and logical way (with the exception of some figures, see below), and the experiments are exhaustive, well-thought and well-described.

Author response:

We thank Reviewer 3 for their careful reading of our manuscript and their comments which we believe helped to improve our manuscript.

Part II – Major Issues: Key Experiments Required for Acceptance

Point 2. A figure that summarizes the findings and represents the proposed entry model, indicating the timing of events (maybe on a separate panel) would be very useful to the readers.

Author response:

We would like to thank Reviewer 3 for this excellent suggestion. We now provide a graphical summary in Figure 10 with the proposed model for TOSV entry into cells.

Point 3. Figure legends need to be more succinct and to contain only the relevant methods info. Most of the figure legend text in the current version would be more appropriate for MM.

Author response:

To take account of this recommendation, we have shortened the figure legends so that they contain only the relevant information on the methods. In this way, we hope to have improved the clarity and readability of the legends.

Point 4. Lines 180-181: “Time-course analysis of the generation of trypan blue-resistant fluorescence of cell-associated TOSV-AF488 revealed that internalization into A549 cells started within the first 5 min and increased over time to reach the half-maximal level (t1/2) within 9+/-2 min and the plateau 10 min later (Fig. 2I).” While the internalization between minutes 20-40 does not increase at the same rate as during first 20 minutes, the number of data points on Figure 2I are not sufficient to support the statement that the plateau is reached at 20 minutes. They rather indicate a slower increase.

Author response:

We monitored TOSV internalization over longer periods (up to 50 min) and included an additional measurement after the first 30 min. We present the results in Figure 2I. This new series of experiments enabled us to determine the kinetics of TOSV internalization with greater accuracy. The text in the Results section has been reworded to take into consideration these new results, in lines 185-188, as follows:

“*Time-course analysis of the generation of trypan blue-resistant fluorescence of cell-associated TOSV-AF488 revealed that internalization into A549 cells started within the first 5 min and increased over time to reach the half-maximal level (t1/2) within 10 ± 2 min and the plateau within 10-15 min later (Fig. 2I).*”

Point 5. Figure 6 – the sequence alignment on panel A should be included in the SI using the font size that can be read without magnifications. The choice of yellow color for RVFV on panels D and E is not the best one. I would suggest grey, or another color that stands out better on the white background. On panels D and E it would be helpful to have the two structures next to each other in addition to the superposition, because it is difficult to visualize the described rotation. Domains I, II and III should be labeled on panels D and E, and a magnified view at domain I to depict where the strands A0B0 and C0D0 located, and how they are different from the RSFV.

Author response:

The sequence alignment of the TOSV and RVFV M segments now uses a letter code for amino acids and is shown in Figure S3. On the other hand, we feel it is important to keep panel A as it was in Figure 6, which has become Figure 7 in the new version of the manuscript. We tried gray color to represent RVFV Gc, but it proved difficult to distinguish from the blue used for TOSV Gc. We, therefore, decided to use the orange color for RVFV in Figures 7D and 7E. Furthermore, both structures of TOSV and RVFV Gc are now shown next to each other in

addition to the superposition in Figures 7D and 7E. The color code used to highlight domains I, II, and III in Figures 7D and 7E is the same as that used in Figures 7B and 7C. Finally, we have added a box in Figure 7E showing a magnification of where the TOSV Gc strands A_0B_0 and C_0D_0 are located and how they differ from those of RVFV Gc.

Point 6. Could the authors provide an explanation as to why TOSV infections of AD549 (Figure 7A) vs BHK21 cells (Figure 7D) show different trends – more or less around 100% in A549 cells at all pH values, and going from 20% at pH 5 to 100% at pH 7.5 in BHK cells? Also, was it possible to infect A549 cells (instead of BHK21) with SFV and UUKV to have a fairer comparison?

Author response:

Reviewer 3 is correct. A549 cells are poorly sensitive to infection with UUKV and SFV. That is why we used BHK-21 cells for these two viruses. We added TOSV to our tests with BHK-21 cells precisely to obtain a fairer comparison. In the experiments shown in Figure 7D, TOSV served as a control for SFV and UUKV. Although BHK-21 cells are used to produce the virus, they are not the most relevant for studying TOSV entry, *i.e.*, BHK-21 cells are not derived from human tissue but from Syrian golden hamsters. Small rodents, on the other hand, are thought to contribute to the reservoirs of the other two arboviruses. To date, we can only speculate as to why TOSV infection is reduced in BHK-21 cells compared with A549 cells. We suspect that the lipid composition, ion fluxes, biochemical properties of cell-surface receptors, or any other cellular factors in hamster cells differ from those in human cells, which could interfere with virus binding and/or the fusion process itself. Nevertheless, 20% of TOSV infectivity remained after exposure to pH 5.5, whereas SFV inactivation is virtually complete at this pH. This point is now discussed in lines 361-363, as follows:

"*We noted that although TOSV infectivity appeared to be lower in BHK-21 cells after exposure to pH ~5.5, 20-30% of virions remained infectious, whereas SFV inactivation was virtually complete (Fig. 8D).*"

Part III – Minor Issues: Editorial and Data Presentation Modifications

Point 7. Figure 1 – could the graphs on C and E panels be grouped into a single panel that contains the label 'neurons' to designate the type of cells used, while the graphs on D and F could similarly be grouped into a single panel with a label 'A549 cells'

Author response:

The graphs in Figures 1C and 1E have been combined in a single panel with the label "Neurons". We labeled panels D and F with "A549 cells" but did not combine the two panels. To follow Point 5 of Reviewer 1, we present the data from Figures 1C to 1F in a separate figure, now Figure 5. We tried, but found that combining the four graphs from panels D and F in a single panel in this new figure results in a loss of clarity.

Point 8. Figure 2 – the white dots are really hard to see on the printed version of the Figure. Could the panels D and F be enlarged or contrast chosen to show the dots better?

Author response:

We assume that Reviewer 3 is referring to panels D and G in Figure 2. The contrast has been enhanced to better highlight the individual dots corresponding to viral particles.

Point 9. Figure 3 – adding labels 'EE', 'LE', and 'Lysosome' to panels B, D, and F, respectively, would orient the reader who is not familiar with the markers of different endosomal compartments (or include that info in the figure legend?)

Author response:

The graphs in Figures 3B, 3D, and 3F are now shown with the labels "Early endosomes", "Late endosomes", and "Late endosomes/lysosomes", respectively.

Point 10. Figure 4 – this figure is very difficult to follow. I suggest grouping the panels by the type of cells or consolidating the panels by some other criteria. For example, graphs D, F, and H could be in the same panel (neurons), and graphs E, G, I and J could be in another panel (A594 cells).

Author response:

The graphs in Figures 4D, 4F, and 4H and Figures 4E, 4G, 4I, and 4J have been combined in a single panel with the label "Neurons" and "A549 cells", respectively, now shown in Figures 4D and 4E.

Point 11. Figure 5 – there is no call for panel A in the manuscript. The titles of Y axes on panels B, C, E and F should not include the (% of XX min / C) – this is confusing and could be explained in the legend. Or it could be changed into "% of the infected cells relative to the 80 min data point" (which becomes too long…).

Author response:

Figure 5A was referenced in line 237 of the original manuscript, and is now referenced as Figure 6A in line 251. The y-axis legends in panels B, E, and F have been changed to "Infected A549 cells (% of control)" and that in panel C to "Infected neurons (% of control)". The samples to which the reference control refers are indicated in the figure legends.

Point 12. Figure 7 legend: Could the description of panels B-E be changed to be easier to follow? Alternatively label the type of cell and virus directly on the graph and skip the explanation in the figure legend

Author response:

This figure is now numbered 8. The upper part of graphs in panels B to E have been labeled with virus and cell type.

Point 13. Line 143 – it should be noted how the measured length of the protrusions compares to the length of the predicted pre-fusion Gc (figure 6C).

Author response:

The protrusions are formed by heterodimers of the two envelope glycoproteins Gn and Gc, which together form pentamers or hexamers composed of 5 or 6 Gn/Gc heterodimers (Halldorsson et al., 2018, Nat Com). The modeling carried out with AlphaFold concerns only the Gc glycoprotein and cannot, therefore, be directly compared with the protrusions.

Point 14. Lines 150-151: "The unbound virions were washed away, and the remaining cell associated, fluorescent viral particles were determined with a fluorimeter" – the sentence is missing a full subject. What was determined – the amount, number, % of fluorescent viral particles…?

Author response:

We apologize for the bad phrasing. The sentence has been corrected and now appears in lines 156-157, as follows:

"*The unbound virions were washed away, and the total fluorescence associated with cell-bound viral particles was determined using a fluorimeter.*"

Point 15. Lines 225-226: Could the authors mention why taxol was not tested on neurons?

Author response:

Access to pluripotent cells and their long period of culture and differentiation into neurons, combined with the limited number of cells produced and available for our tests, led us to prioritize the experiments with iPSC-derived neurons, and consequently to limit the number of inhibitors to be tested with these cells. As taxol did not affect TOSV infection in A549 cells at all, this inhibitor was not considered a priority and was therefore not tested.

Point 16. Lines 242 – 288: This section needs some polishing to improve the clarity.
a. For example, line 246 "Virus entry was then synchronized by rapid warming..." – could it be explained why this synchronizes the virus?

b. Line 248 – "…concentration of 50 mM was used to ensure that infection …" – instead say "NH4Cl was added to 50mM to ensure …".
c. Figure 5A is not mentioned.
d. Lines 273-275: Calls for figure should be made at appropriate places instead at the end of the sentence. The sentence should be modified to: "At 25 °C, fusion corresponded to 40% of the 37 °C control (Figure 5F), whereas infection via the normal route was lowered by 85% (Figure 5E).
e. Lines 278-298: In the sentence "Most likely the viral particles did not infect at 25 °C and below because they did not reach a compartment with low enough pH." – does the "compartment with low enough pH" correspond to LE? If yes, why not be specific and use 'LE' instead?

Author response:

a. The attachment of viral particles to cells on ice allows for virus binding, but not internalization. Once unbound viruses have been washed away and the temperature subsequently warmed, bound viral particles undergo internalization at the same time. The whole process is known as "synchronization". We have reworded the sentences in lines 155-156, 177-181, and 259-261 for greater clarity, as follows:

"*Incubation on ice allows virions to bind to the cell surface but prevents their internalization into cells.*"

"*To analyze TOSV internalization by fluorescence microscopy, we synchronized virus entry. To this end, we first allowed TOSV-AF488 to bind to A549 cells on ice at high MOI (~10). The cells were washed to remove unbound viruses, then rapidly warmed to 37 °C to trigger endocytosis, and finally placed back on the ice after 30 min to stop further endocytosis.*"

"*To synchronize virus entry, virions were first allowed to bind to neurons and A549 cells on ice at MOIs of 15 and 1, respectively. Virus entry was then allowed by rapid warming to 37 °C, and NH_4Cl was added at different times following the temperature switch.*"

b. To avoid repetition of the word "added" in two successive sentences, lines 260-261, this sentence reads, in lines 262-263, as follows:

"*NH_4Cl was used at 50 mM to ensure that infection was completely abolished after adding the weak base.*"

c. Please, see our response to Point 11 of this Reviewer.

d. The sentence now reads, in lines 287-289, as follows:

"*At 25 °C, fusion corresponded to 40% of the 37 °C control (Fig. 6F), whereas infection via the normal route was lowered by 85% (Fig. 6E).*"

e. The intraluminal pH value varies in LEs according to their degree of maturation, and strictly speaking, there is not just one category of LEs but several. This sentence now reads, in lines 292-293, as follows:

"*Most likely the viral particles did not infect at 25 °C and below because they did not reach LEs with low enough pH.*"

Point 17. Lines 321-323: Could the authors elaborate more on this interesting statement reported in reference 30?

Author response:

We thank Reviewer 3 for their interest in this most intriguing molecular distinction in the *Phenuiviridae* family, but we feel that our data are limited in this regard and do not support a more elaborate discussion.

Point 18. Line 364 – several time the expression 'we forced … fusion' is used in the manuscript. Choice of 'induced' instead of 'forced' might be better?

Author response:

The term "forced" has been replaced by "induced" in lines 378-380, as follows:

"*To further examine the effect of pH on TOSV activation and fusion, we induced TOSV-R18 fusion on the A549 cell surface with buffers ranging in pH from 5.0 to 5.8 as described above and monitored the dye dequenching in real time.*"
