# Supplementary material for: The phenuivirus Toscana virus makes an atypical use of vacuolar acidity to enter host cells
Source: PLoS Pathog. 2023 Aug 14;19(8):e1011562. doi: 10.1371/journal.ppat.1011562 (PMC10449198; doi:10.1371/journal.ppat.1011562)
Supplement: Attachment — Submitted filename: Review PLOS Path.doc [file ppat.1011562.s011.doc]

The authors report new elements concerning the mechanisms of entry and fusion of the Toscana Virus in iPSC-derived neurons and in different cell lines. The manuscript is interesting and several experiments have been carried out to provide a valid hypothesis on how binding internalization and membrane fusion can occur during TOSV infection.

The rationale of this study is clear; the methodology pursued in this study is accurate and well-conducted. The results are helpful to understand the mechanisms by which TOSV, as well as other members of *Phleboviridae*, enter and infect host cells.

Specific points for consideration:

**Major points**

It is not mentioned that the virus (TOSV) was plaque-purified, and it was collected from BHK-21 infected cells. The fact that most of the infecting virus is not infectious could be due to the presence of a lot of defective particles present in the viral stock. Although it is known that virions can have even less than 3 genomic RNA segments, it would be useful to work with a rather pure viral stock, that was purified on sucrose gradient, but the band could be contaminated with defective particles.

Discussion: the results of the experiments are sometimes reported in a not well organized way, therefore the reader may lose the thread. This is perhaps due to the amount of data the authors need to comment on. It appears that the acidic environment of the EEs is useful for the entry of TOSV into the cytosol, but LES are crucial for infection, therefore the role of both Endosomes should be better explained. I would suggest to make it clearer, trying to better schematize the path of the virus inside the cell until fusion.

**Minor points**

- Besides 1 and 2, there are more specific references to be added describing most severe cases of neurologic infections by TOSV.

-Lines 94: it would be better to specify the three sandfly cell lines used, although mentioned in Mat & Meth.

-Lines 239-240: ‘Additional processing…’ is a little bit confusing for the reader, since the glycoproteins have a role in the fusion.

Line 474: please write the full form of the acronym ‘L-PVs’, as it is mentioned for the first time, and use the acronym from then on.

- Is Ref. 53 missing?
